# *BrWRKY8*: a key regulatory factor involved in delaying postharvest leaf senescence of Pakchoi (*Brassica rapa* subsp. *chinensis*) by 2,4-epibrassinolide

**DOI:** 10.1093/hr/uhaf004

**Published:** 2025-01-06

**Authors:** Xuesong Liu, Yinghao Xu, Yujun Zhang, Xiaofei Chen, Pengxia Li

**Affiliations:** Institute of Agricultural Facilities and Equipment, Jiangsu Academy of Agricultural Sciences, 50 Zhongling Road, Nanjing 210014, Jiangsu, China; Department of Food Science, Shenyang Agricultural University, 120 Dongling Road, Shenyang 110043, China; Department of Food Science, Shenyang Agricultural University, 120 Dongling Road, Shenyang 110043, China; Department of Food Science, Nanjing Agricultural University, 666 Binjiang Road, Nanjing 211800, China; Institute of Agricultural Facilities and Equipment, Jiangsu Academy of Agricultural Sciences, 50 Zhongling Road, Nanjing 210014, Jiangsu, China; Department of Food Science, Shenyang Agricultural University, 120 Dongling Road, Shenyang 110043, China; Jiangsu Key Laboratory for Horticultural Crop Genetic Improvement, 50 Zhongling Road, Nanjing 210014, Jiangsu, China; Key Laboratory of Cold Chain Logistics Technology for Agro-Products, Ministry of Agriculture and Rural Affairs, 50 Zhongling Road, Nanjing 210014, China

## Abstract

Brassinosteroids (BRs) are extensively distributed in plants and play crucial roles throughout all stages of plant growth. Nevertheless, the molecular mechanism through which BRs influence postharvest senescence in pakchoi remains elusive. Previous studies have demonstrated that the application of 1.5 μM of the BRs analog 2,4-epibrassinolide (EBR) delayed the leaf senescence in harvested pakchoi. In this study, we constructed the EBR-delayed senescence transcriptome in pakchoi leaves and discovered that EBR modulates the expression of genes involved in the chlorophyll (Chl) metabolism pathway and the BRs pathway in pakchoi. Notably, we identified and characterized an EBR-suppressed, nucleus-localized WRKY transcription factor called *BrWRKY8*. *BrWRKY8* is a highly expressed transcriptional activator in senescent leaves, targeting the promoters of the Chl degradation-associated gene *BrSGR2* and the BRs degradation-associated gene *BrCHI2*, thereby promoting their expression. Overexpression of the *BrWRKY8* gene accelerated the senescence process in *Arabidopsis* leaves, while EBR treatment mitigated the leaf senescence phenotype induced by *BrWRKY8* overexpression. Conversely, silencing of *BrWRKY8* through the virus-induced gene silencing extended the postharvest storage period of pakchoi. In conclusion, the newly discovered BRs-*BrWRKY8* regulatory model in this study provides novel insights into BRs-mediated leaf senescence in pakchoi.

## Introduction

Senescence denotes to the natural deterioration of a specific organ or the entire plant within a plant body and represents the final stage in a plant’s life cycle [1]. This complex biological process involves the redistribution of organic matter within the plant [2]. Leaf senescence, a form of plant cell death, is not a passive degenerative process but rather the result of orderly regulation by specific internal plant factors. It encompasses various internal factors that govern the systematic degradation of cellular components, leading to decreased metabolic activity, cell death, and protein breakdown [3]. Senescence serves as a mechanism through which plants respond to environmental changes and adapt to fluctuating conditions [4]. However, abnormal or premature senescence can adversely affect postharvest quality and increase losses in horticultural vegetables [5]. Consequently, elucidating the molecular mechanisms underlying postharvest senescence and yellowing in leafy vegetables is crucial for maintaining postharvest quality, extending shelf life, and minimizing losses.

BRs are crucial plant hormones that enhance plant growth and regulate various physiological and biochemical functions [6]. The biosynthesis of BRs is meticulously controlled by a series of enzymes [7]. Several enzymes involved in catalyzing Brassinosteroid (BR) synthesis have been identified. For instance, DE-ETIOLATED2 (DET2), a 5-α reductase, catalyzes the 5-α reduction reaction from Noguchi to campestanol [7]. Furthermore, multiple cytochrome P450 oxidases participate in BR synthesis, including constitutive photomorphogenesis and dwarfism (CPD), dwarf4 (DWF4), rotundifolia3 (ROT3), brassinosteroid-6-oxidase 1 (BR6ox1), and BR6ox2 [8]. Conversely, BR degradation represents a significant pathway for regulating endogenous BRs levels. In *Arabidopsis*, this degradation is primarily facilitated by two cytochrome P450 monooxygenases, phyB activation-tagged suppressor1 (BAS1) and chibi2 (CHI2), which promote the breakdown of biologically active BR [9].

The role of BRs in leaf senescence is complex, and the underlying mechanisms remain incompletely understood. Evidence suggests that BRs may function as positive regulators of senescence. For example, a mutant in *Arabidopsis* that affecting BR biosynthesis, known as de-etiolated 2 (*det2*), exhibits delayed leaf yellowing [7]. However, BRs are also widely recognized as plant hormones that can delay postharvest storage and preservation [10]. EBR, a synthetic BR with high activity, is commonly used in research. The influence of EBR on broccoli yellowing are concentration-dependent, with 2 μM EBR treatment effectively delaying yellowing [11]. Additionally, EBR application has been shown to enhance the antioxidant capacity of broccoli, leading to improved control of postharvest yellowing [12]. In our previous research, we observed that treatment with 1.5 μM EBR significantly inhibited leaf senescence in harvested pakchoi [13]. Nevertheless, the specific molecular mechanisms and transcription factors (TFs) involved in the EBR-mediated delay of leaf senescence in pakchoi remain poorly understood.

The regulation of senescence involves a complex network of hormonal signaling, TFs, and metabolic enzymes [14, 15]. The WRKY TF family plays a significant role in hormone-regulated leaf senescence [16, 17]. In *Arabidopsis*, *AtWRKY75* regulates ethylene-mediated leaf senescence through abscisic acid (ABA) signaling and interacts with other TFs and proteins in pathways involving jasmonic acid (JA), auxin, salicylic acid (SA), and ethylene-mediated leaf senescence [18, 19]. *AtWRKY57* interacts with indoleacetic acidindoleacetic acid 29 (*IAA29)* and jasmonate ZIM-domain 4/8 (*JAZ4/8*) to co-regulate JA and growth hormone-mediated leaf senescence [20]. *AtWRKY45* interacts with RGA-LIKE1 (*RGL1*) through the gibberellic acid (GA) signaling pathway, thereby regulating leaf senescence [21]. The signaling cascade formed by nonexpressor of pathogenesis-related genes 1(*NPR1*), *WRKY46*, and *WRKY6* is crucial in the SA- and probenazole-mediated leaf senescence and Chl degradation process [15]. In rice, *OsWRKY80* is positively regulated by ABA and promotes leaf senescence [22], while *OsWRKY42* inhibits the *OsMT1d*-mediated reactive oxygen species (ROS) scavenging pathway, thereby promoting leaf senescence [23]. Similar findings have been reported in horticultural crops, where WRKY TFs contribute to fruit and vegetable senescence. For instance, in Chinese cabbage, *BrWRKY6* regulates GA to inhibit leaf senescence, whereas *BrWRKY65* activates gene expression related to Chl degradation and positively regulates senescence [24, 25]. However, the specific roles of WRKY TFs in EBR-mediated leaf senescence in pakchoi remain to be elucidated.

Pakchoi, a leafy vegetable widely consumed in China, is valued for its high nutritional content [26]. However, its storage and shelf life are compromised by postharvest quality deterioration, including yellowing and decay. Consequently, elucidating the mechanisms underlying postharvest yellowing and senescence in pakchoi is essential for extending its shelf life. This study aimed to investigate the intrinsic molecular mechanisms of WRKY TFs in EBR-mediated retardation of postharvest pakchoi leaf senescence. Our research demonstrates that EBR modulates the expression of genes associated with Chl metabolism and the BR pathway. We have identified *BrWRKY8* as a key EBR-regulated TF that promotes leaf senescence by activating specific gene transcription. This investigation provides novel insights into the transcriptional regulation of WRKY TFs involved in EBR-mediated leaf senescence, enhances our understanding of leaf senescence mechanisms, and presents opportunities for improving postharvest techniques in pakchoi preservation.

## Results

### EBR delays leaf senescence in pakchoi

**Figure 1:**
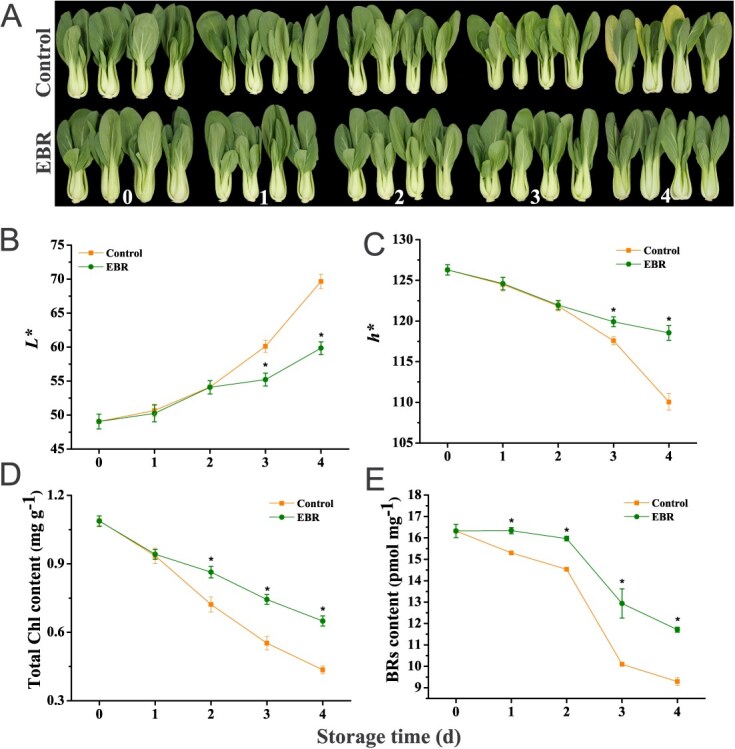
Forty-day-old pakchoi specimens were subjected to 1.5 μM EBR treatment. (**A**) Phenotypes of harvested pakchoi with and without EBR treatment (0–4 d). Scale bar = 2 cm. L* value (**B**), h* value (**C**), and concentrations of total Chl (**D**) and BRs (**E**) in pakchoi stored at 20°C. Data represent means ± standard deviation of three biological replicates. Vertical bars indicate standard deviation. Asterisks denote significant differences at *P* < 0.05

To elucidate the influence of EBR on the storage period of postharvest pakchoi, we treated the plants with 1.5 μM EBR and stored them at approximately 20°C in dark conditions. Starting from the third day, the control group exhibited leaf yellowing, while the EBR-treated group remained free of yellowing throughout the storage period (Fig. 1A). Surface color L* values increased in all treatment groups during storage, with the control group demonstrating higher L* values than the EBR treatment group during the third and fourth days (Fig. 1B). The surface color h* values showed a decreasing trend throughout the storage period, with the EBR-treated group maintaining higher h* values compared to the control group on the third and fourth days of storage (Fig. 1C). Additionally, we determined the total Chl and BRs content in each sample. During storage days 2–4, the EBR-treated group had higher total Chl content in comparison with the control group (Fig. 1D). As the storage duration increased, the content of BRs showed a decreasing trend, with the EBR-treated group demonstrating higher levels of BRs compared to the control group from the first to fourth day of storage (Fig. 1E).

### Transcriptome analysis of EBR-delayed leaf senescence in pakchoi

To further elucidate the effect of EBR on postharvest leaf senescence in pakchoi, we conducted transcriptome sequencing and analysis. We examined pakchoi leaves from the control at 0 days (CK0), the EBR-treated group at 4 days (EBR4), and the control at 4 days (CK4), with three replicates for each treatment. The sequencing data yielded high-quality results, with over 99% of reads exceeding quality thresholds (Supplementary Table S2). The average GC content was approximately 48.1%, and the Q30 base percentage exceeded 94.18% in each library, indicating reliable data (Supplementary Table S3). By aligning the clean reads to the ‘Brara Chiifu’ genome database, we identified 48 319 expressed genes, including 2069 novel genes (Supplementary Table S4). More than 87% of the clean reads aligned to the genome, confirming the suitability of ‘pakchoi’ genome as a reference (Supplementary Table S5). The fragments per kilobase million (FPKM) values reflected gene expression levels in the transcriptome, with numerous genes exhibiting log_10_ (FPKM) values greater than 0 (Supplementary Fig. S1A). Additionally, Pearson’s correlation coefficients exceeded 0.95, indicating strong correlation between replicates (Supplementary Fig. S1B). These results demonstrate the high quality of the transcriptome libraries, supporting their use in subsequent experimental analysis.

**Figure 2:**
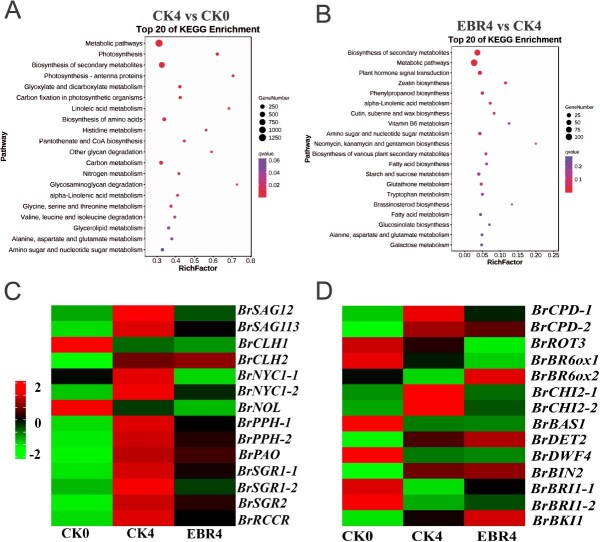
Transcriptome analysis of pakchoi under 1.5 μM EBR treatment. KEGG enrichment analysis of DEGs for CK4/CK0 (**A**) and EBR4/CK4 (**B**). (**C** and **D**) Expression profiles of Chl metabolism, senescence, and BR-associated genes in the transcriptome following EBR treatment of pakchoi. The average FPKM values in each sample responding to EBR at 0 and 4 days of treatment were used to generate the heatmap

Differentially expressed genes (DEGs) were selected from the libraries using fold change ≥2 and false discovery rate (FDR) ≤ 0.01 as the screening criteria. Through comparative analysis of the transcriptomes from the three treatment groups, we identified 10 032 (CK0/CK4), 9872 (CK0/EBR4), and 931 (CK4/EBR4) DEGs (Supplementary Fig. S2A). From these, 269 overlapping DEGs between CK0/CK4 and CK4/EBR4 were selected for further examination (Supplementary Fig. S2B). Heat map clustering analysis was performed on all DEGs in the CK0/CK4 and CK4/EBR4 groups (Supplementary Fig. S2C and D). The Kyoto Encyclopedia of Genes and Genomes (KEGG) enrichment analysis disclosed remarkable enrichment in metabolic pathways, biosynthesis of secondary metabolites, and photosynthesis pathways in pakchoi after four days of storage (Fig. 2A). The EBR-treated group exhibited significant enrichment in biosynthesis of secondary metabolites, metabolic pathways, plant hormone signal transduction pathways, and BRs biosynthesis pathway (Fig. 2B). These findings suggest that EBR, as a hormone-signaling molecule, can regulate the expression of genes involved in Chl metabolism, BRs signaling pathway, and BRs biosynthesis pathway, thereby inhibiting the degradation of BRs in leaves. Collectively, these data provide reliable support for subsequent investigations.

The KEGG enrichment analysis screened two senescence-associated genes, 12 Chl metabolism-related genes, and 14 BR-related genes. Among these, *BrSAG12*, *BrSAG113*, *BrCLH2*, *BrNYC1*, *BrPPH*, *BrPAO*, *BrSGR1*, *BrSGR2*, *BrRCCR*, and *BrCHI2* were induced by senescence and inhibited by EBR treatment (Fig. 2C and D). To validate these findings, *BrSAG12*, *BrNYC1*, *BrSGR2*, and *BrCHI2* were selected for quantitative real-time PCR (qRT-PCR) analysis to examine their expression levels in treated and untreated groups over 0 to 4 days of storage. The results revealed that the expression of these four genes was induced to varying degrees with increasing storage duration, and EBR treatment reduced their expression levels (Supplementary Fig. S3A-D).

### *BrWRKY8* exhibits elevated expression during postharvest storage of pakchoi and is inhibited by EBR

**Figure 3:**
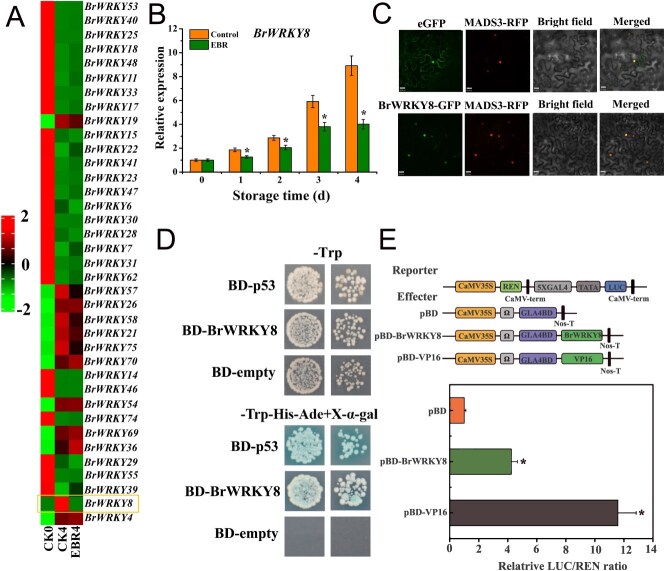
Screening of WRKY TFs in response to EBR and basic characterization of *BrWRKY8* in *Brassica napus* (**A**) Expression profiles of all detected BrWRKY genes in the transcriptome. (**B**) Transcript levels of *BrWRKY8* in leaves of 1.5 μM EBR-treated and untreated pakchoi during storage. (**C**) Tobacco was used to study the subcellular localization of the empty vector (eGFP) and the fusion expression vector (*BrWRKY8*-GFP). (**D**) Detection of *BrWRKY8* transcriptional activity using yeast 2 hybrid (Y2H) yeast. (**E**) Analysis of transcriptional activity of *BrWRKY8* in tobacco. Data represent means ± standard deviation of three biological replicates. Vertical bars indicate standard deviation. Asterisks denote significant differences at *P* < 0.05

Gene ontology (GO) annotation analysis of the DEGs between CK4 and EBR4 in pakchoi revealed a high enrichment of ‘nucleic acid binding TF activity’ in the molecular function category (Supplementary Fig. S4A and B). TF expression levels are known to be strongly associated with plant senescence, and WRKY TFs play crucial roles in various plant-aging processes [17]. In our analysis of the pakchoi genome database and the plant TF database, we identified 180 *BrWRKY* genes in the pakchoi genome. To identify key WRKY TFs that may play significant roles in EBR-delayed senescence in pakchoi, we analyzed the DEGs in the transcriptome of EBR-treated pakchoi leaves. Heatmaps revealed that the expressions of *BrWRKY19*, *BrWRKY57*, *BrWRKY58*, *BrWRKY75*, and *BrWRKY8* were significantly increased after 4 days; however, only the *BrWRKY8* was noticeably inhibited by EBR treatment (Fig. 3A).

The *BrWRKY8* gene spans 3742 bp and comprises three exons (Supplementary Fig. S5A). The WRKY domain of *BrWRKY8* exhibits high conservation across various species (Supplementary Fig. S5B). Three-dimensional structure prediction indicates that *BrWRKY8* initiates with a WRKYGQK motif at the N-terminus and incorporates four consecutive β-sheet domains (Supplementary Fig. S5C). To elucidate the evolutionary relationship of *BrWRKY8*, we constructed a phylogenetic tree using pakchoi and *Arabidopsis* WRKYs. Supplementary Fig. S5D illustrates that *BrWRKY8* clusters with *BrWRKY10* and *BrWRKY12*. Additionally, to further explore the role of *BrWRKY8* in leaf senescence, its expression pattern was analyzed in senescent leaves stored for 4 days. The senescent leaves were divided into three regions: completely yellowed (bottom), partially yellowed (middle), and nonyellowed (top) (Supplementary Fig. S5E). The expression of *BrWRKY8* was significantly higher in the completely yellowed area compared to other parts (Supplementary Fig. S5F), suggesting that *BrWRKY8* may play an important role in the senescence of pakchoi leaves.

Expression analysis of *BrWRKY8* revealed a gradual increase in its transcript level during postharvest storage of pakchoi. However, EBR treatment inhibited the expression of *BrWRKY8* (Fig. 3B). This suggests that *BrWRKY8* may contribute to the delayed leaf senescence observed in pakchoi following EBR treatment. To determine the subcellular localization of *BrWRKY8*, a fusion expression vector of *BrWRKY8* and green fluorescent protein (GFP) was constructed and co-transferred with a nuclear marker into tobacco leaves, with empty vector as control. The results showed that the control had fluorescent signals in all parts of the cell, while the green fluorescent signal of *BrWRKY8* overlapped with the red fluorescent signal of the nuclear marker. This indicates that *BrWRKY8* is a nucleus-localized TF (Fig. 3C). To examine the transcriptional activity of *BrWRKY8*, a fusion expression vector of *BrWRKY8* and the yeast GAL4 DNA-binding domain (BD) was constructed and transformed into yeast cells. The positive control strain containing the BD-p53 fusion expression vector and the BD-*BrWRKY8* strain both exhibited normal growth and α-galactosidase activity on selective medium, while the negative control strain containing the empty BD vector failed to survive on this medium (Fig. 3D). These results suggest that *BrWRKY8* functions as a transcriptional activator. The transcriptional activation of *BrWRKY8* was further validated in tobacco using dual-luciferase reporter (DLR) assays. The luciferase/renilla luciferase (LUC/REN) ratios of both BD-*BrWRKY8* and the positive control VP16 were 4.2-fold and 11.6-fold higher, respectively, than that of the control, further confirming the transcriptional activation ability of *BrWRKY8* (Fig. 3E).

### *BrWRKY8* activates *BrSGR2* and *BrCHI2* transcription by binding to their promoters

**Figure 4:**
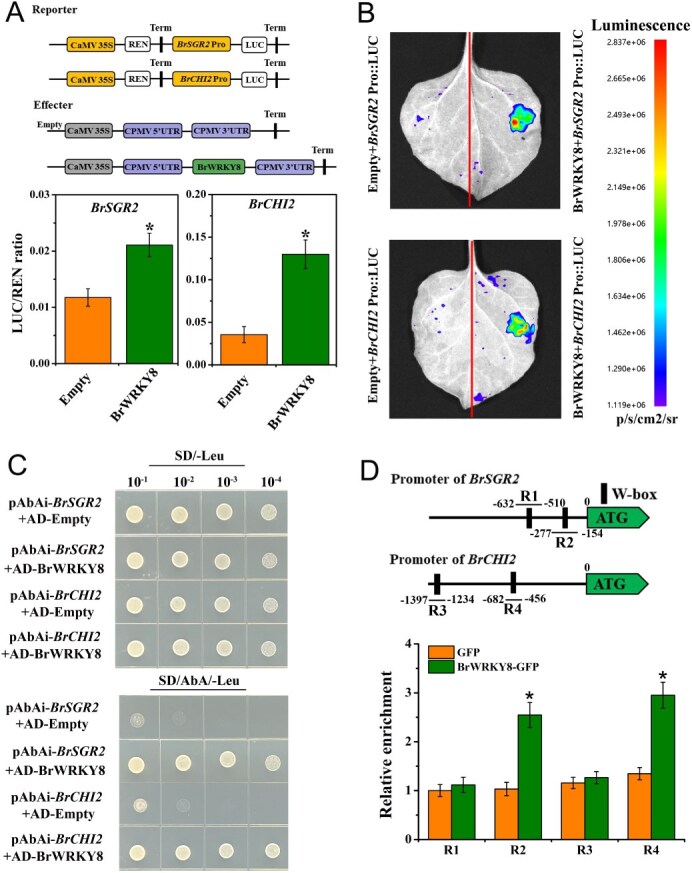
*BrWRKY8* binds to the *BrSGR2* and *BrCHI2* promoters. (**A**) LUC/REN ratio of *BrWRKY8* after interaction with promoters (*BrSGR2* pro and *BrCHI2* pro) in vivo. (**B**) Luciferase fluorescence images of *BrWRKY8* co-transformed with *BrSGR2* and *BrCHI2* promoters in tobacco leaves. (**C**) Y1H assays. pAbAi-*BrSGR2* + AD-*BrWRKY8* and pAbAi-*BrCHI2* + AD-*BrWRKY8* represent the experimental group. pAbAi-*BrSGR2* + AD-Empty and pAbAi-*BrCHI2* + AD-Empty serve as the respective negative controls. (**D**) Schematic representation of ‘W-box’ in *BrSGR2* and *BrCHI2* promoters. ChIP-qPCR assay demonstrating *BrWRKY8* binding to the *BrSGR2* and *BrCHI2* promoters in pakchoi. ChIP assays were performed with chromatin prepared from *BrWRKY8*-GFP transient transformation plants, using an anti-GFP antibody (IP). Data represent means ± standard deviation of three biological replicates. Vertical bars indicate standard deviation. Asterisks denote significant differences at *P* < 0.05

WRKY TFs are known to regulate the expression of target genes by binding directly to the ‘W-box’ motif (consensus sequence: (T/C)TGAC(C/T)) in the promoters of downstream genes [18]. In this study, we observed similar transcriptional patterns of *BrWRKY8* and several other genes including *BrSAG12*, *BrSAG113*, *BrCLH2*, *BrNYC1*, *BrPPH*, *BrPAO*, *BrSGR1*, *BrSGR2*, *BrRCCR*, and *BrCHI2* (Fig. 2C and D and Fig. 3A). Based on this observation, we hypothesized that *BrWRKY8* might regulate the transcriptional expression of these genes by binding to the ‘W-box’ motif in their promoters. To test this hypothesis, we analyzed the promoter regions (2-kb) of the above 10 genes and found that they contained more than one ‘W-box’ motif (Supplementary Fig. S6). The results of the DLR experiments showed that the *BrWRKY8* overexpression vector (35S::*BrWRKY8*), when co-expressed in tobacco with the *BrSGR2* and *BrCHI2* promoters (*BrSGR2* Pro::LUC and *BrCHI2* Pro::LUC), led to an increased LUC/REN ratio compared with the control (Fig. 4A), and no difference in LUC/REN ratios was observed with controls after mutations in the *BrSGR2* and *BrCHI2* promoter binding sites (*BrSGR2* Pro-m and *BrCHI2* Pro-m) (Supplementary Fig. S7). Furthermore, the fluorescence signal intensity produced by *BrWRKY8* interacting with the promoters of *BrSGR2* and *BrCHI2* was also higher than that of their respective controls (Fig. 4B). However, *BrWRKY8* co-expressed in tobacco with the remaining eight gene promoters showed little change in the LUC/REN ratio compared to the control (Supplementary Fig. S8A and B).

To further validate the binding ability of *BrWRKY8* to the promoters of *BrSGR2* and *BrCHI2*, a yeast one-hybrid (Y1H) assay was conducted. In this assay, *BrWRKY8* served as the prey. First, the inhibitory concentrations of aureobasidin A (AbA) were screened to suppress transcriptional self-activation from two promoters, pAbAi-*BrSGR2* and pAbAi-*BrCHI2*. The lowest inhibitory concentration was determined to be 200 ng ml^−1^. At this background concentration, yeast strains containing pAbAi-*BrSGR2* + pGADT7 (AD)-*BrWRKY8* and pAbAi-*BrCHI2* + AD-*BrWRKY8* exhibited normal growth on synthetic defined (SD)/−Leu/AbA medium, indicating an interaction between *BrWRKY8* and the promoters of *BrSGR2* and *BrCHI2*. Conversely, the control strains containing pAbAi-*BrSGR2* + AD-Empty and pAbAi-*BrCHI2* + AD-Empty failed to grow on SD/−Leu/AbA medium (Fig. 4C).

To further validate the binding capacity of *BrWRKY8* to the *BrCHI2* and *BrSGR2* promoters in pakchoi, chromatin immunoprecipitation (ChIP) was conducted. Initially, transient overexpression of the GFP empty vector and the *BrWRKY8*-GFP fusion vector in pakchoi was performed. Subsequently, ChIP was executed using a specific GFP antibody. The quantity of immunoprecipitated chromatin was determined by qPCR on distinct regions of *BrSGR2* and *BrCHI2*. Four fragments (R1-R4) containing the ‘W-box’ motifs were selected from the promoters of *BrCHI2* and *BrSGR2* for testing. The ChIP-qPCR analysis revealed that both the *BrSGR2* promoter (R2) and *BrCHI2* promoter (R4) fragments were significantly enriched following immunoprecipitation (Fig. 4D). These findings provide further evidence that *BrWRKY8* binds to the promoters of *BrCHI2* and *BrSGR2*.

### *BrWRKY8* overexpression accelerates leaf senescence in *Arabidopsis thaliana*

**Figure 5:**
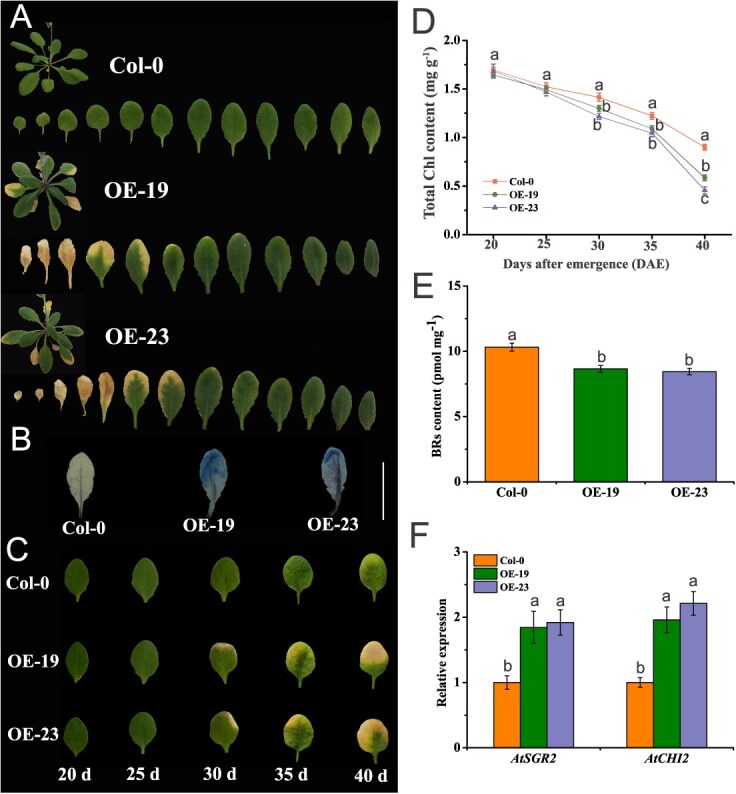
*BrWRKY8* overexpression accelerates leaf senescence in *Arabidopsis*. (**A**) Phenotypes of different lines (Col-0, OE19, and OE23) at 40 days postgermination. (**B**) Dead cells of Col-0, OE19, and OE23 leaves were stained with dye 35 days postgermination, scale bar = 1.5 cm. (**C**) Phenotype of Col-0, OE19, and OE23 plants at 20, 25, 30, 35, and 40 days postemergence. (**D**) Quantification of Chl content in Col-0, OE19, and OE23 plants at 20, 25, 30, 35, and 40 days postemergence. (**E**) Measurement of endogenous BRs levels in different lines at 14 days postemergence. (**F**) Analysis of *AtCHI2* and *AtSGR2* expression levels in different lines at 14 days postemergence. Data represent means ± standard deviation of three biological replicates. Vertical bars indicate standard deviation. The distinct letters suggest significant differences at *P* < 0.05

To further elucidate the role of *BrWRKY8* in leaf senescence, several independent lines overexpressing *BrWRKY8* were screened and characterized. Two lines (OE19 and OE23) were randomly selected for further analyses (Supplementary Figure S9). These two overexpression lines were cultured together with wild-type (Col-0) under suitable conditions. At 35 days after germination, the first to sixth leaves of the OE19 and OE23 lines exhibited severe yellowing in contrast to Col-0 (Fig. 5A). To further assess leaf senescence, leaves from each line at 35 days underwent Evans blue staining. The results showed that Col-0 leaves had lighter blue spots compared to *BrWRKY8*-OE19 and *BrWRKY8*-OE23 (Fig. 5B). Moreover, examination of the 4th true leaf at various leaf ages (20, 25, 30, 35, and 40 days) revealed that OE19 and OE23 exhibited a higher degree of leaf yellowing than Col-0 between 30 to 40 days (Fig. 5C). Additionally, Additionally, we determined the Chl content of 4th true leaf of Arabidopsis at 20, 25, 30, 35, and 40 days postgermination, and the results showed that overexpression lines had lower total Chl content than Col-0 lines at days 30, 35, and 40 (Fig. 5D). These findings suggest that *BrWRKY8* may play a crucial role in regulating plant senescence.

To investigate the regulatory mechanism of *BrWRKY8* in promoting leaf senescence, the expression levels of *AtCHI2* and *AtSGR2* (homologues of *BrCHI2* and *BrSGR2*) were analyzed in this study. Initially, the expression of *AtSGR2* and *AtCHI2* was monitored in Col-0 from 20 to 35 days of leaf age, revealing an increase in expression levels correlating with leaf age (Supplementary Figure S10). Quantitative RT-PCR analysis demonstrated elevated expression levels of *AtSGR2* and *AtCHI2* in OE19 and OE23 lines compared to Col-0 (Fig. 5E). Additionally, measurement of endogenous BRs levels in the transgenic lines indicated lower concentrations in the overexpressing plants relative to Col-0 (Fig. 5F). These findings suggest that *BrWRKY8* may enhance expression of the BRs degradation gene *AtCHI2* and the Chl metabolism gene *AtSGR2,* potentially contributing to the intensified leaf yellowing phenotype observed in *Arabidopsis*.

**Figure 6:**
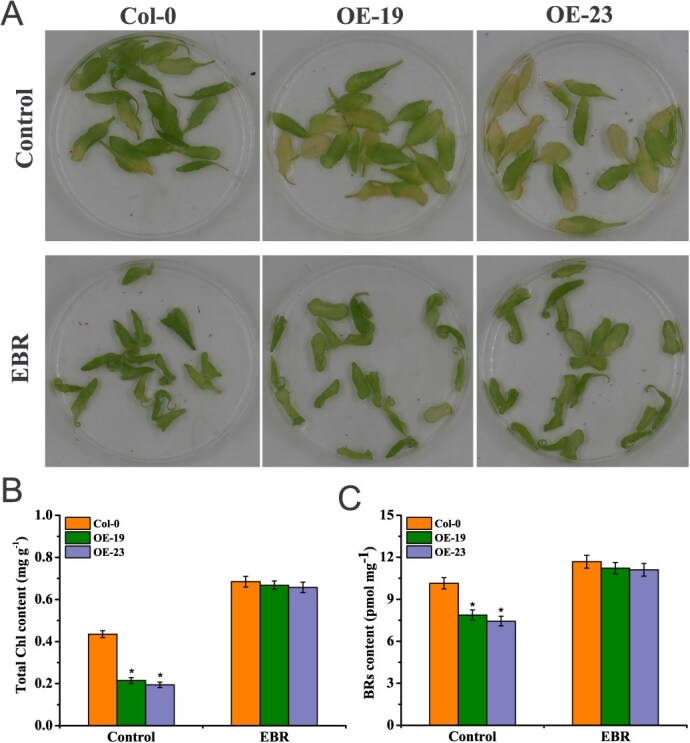
The seventh and eighth leaves of three-week-old *Arabidopsis* plants were isolated and subjected to treatment with 1.5 μM EBR under 22°C dark conditions for 4 days. Leaf senescence (**A**), Chl content (**B**), and endogenous BRs content (**C**) were evaluated in comparison to the control group. Data represent means ± standard deviation of three biological replicates. Vertical bars indicate standard deviation. Asterisks denote significant differences at *P* < 0.05

In the absence of treatment, detached leaves from *BrWRKY8*-OE19 and *BrWRKY8*-OE23 exhibited significantly higher chlorosis compared to Col-0. However, upon exogenous application of 1.5 μM EBR, the chlorosis phenotype in the transgenic lines was suppressed, and their total Chl content became comparable to that of Col-0 (Fig. 6A and B). Moreover, analysis of BRs content revealed higher levels in Col-0 compared to the transgenic lines. EBR treatment, however, increased the endogenous BRs content in both Col-0 and the transgenic lines, effectively eliminating the difference in BRs content between them (Fig. 6C). These findings confirm the crucial role of *BrWRKY8* in delaying EBR-induced leaf yellowing in *Arabidopsis*.

### Endogenous inhibition of *BrWRKY8* extends postharvest storage of pakchoi

**Figure 7:**
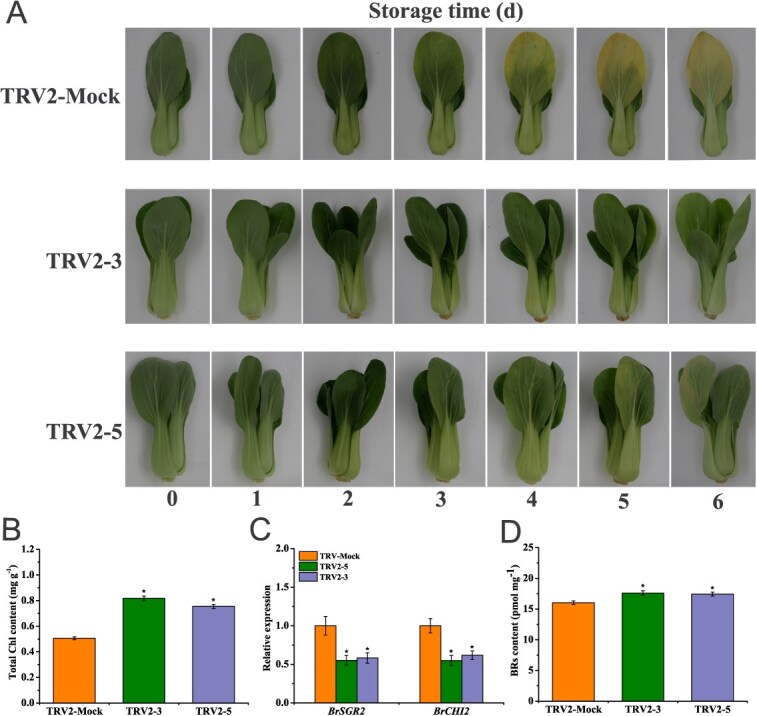
Silencing of *BrWRKY8* prolongs postharvest storage of pakchoi. (**A**) Senescence phenotypes of silenced strains (TRV2-3 and TRV2-5) and control (TRV2-Mock). (**B**) Quantification of total Chl content in TRV2-Mock, TRV2-3, and TRV2-5 plants after 6 d of storage. (**C**) Expression levels of *BrSGR2* and *BrCHI2* were analyzed in TRV2-3, TRV2-5, and TRV2-Mock plants at 30 days postemergence. (**D**) Endogenous BRs levels were measured in *BrWRKY8*-silenced and control plants. Data represent means ± standard deviation of three biological replicates. Vertical bars indicate standard deviation. Asterisks denote significant differences at *P* < 0.05

To further examine the endogenous impact of *BrWRKY8* on pakchoi, we obtained eight lines with silenced *BrWRKY8* expression using VIGS technology. Compared with the control (TRV2-Mock), *BrWRKY8* expression was reduced by 64.6% and 68.5% in TRV2-3 and TRV2-5, respectively, which were selected for the subsequent study (Supplementary Fig. S11). Plants of TRV2-3, TRV2-5, and TRV2-Mock were harvested at 40 days after germination and stored in darkness at 20°C for 6 days. The control TRV2-Mock showed yellowing on the fourth day, whereas TRV2-3 and TRV2-5 showed only slight yellowing on the sixth day, with less yellowing than control throughout the storage period (Fig. 7A). The total Chl content of the plants stored for 6 days showed TRV2-3 and TRV2-5 retained greater Chl levels than TRV2-Mock (Fig. 7B). In order to investigate the mechanism of *BrWRKY8* in regulating postharvest senescence in pakchoi, the expression levels of *BrMYB108* downstream genes *BrSGR2* and *BrCHI2* were analyzed by qRT-PCR, and it was found that the expression of *BrSGR2* and *BrCHI2* in the silenced lines was reduced by 41.8%–44.9% and 38.4%–45.2, respectively, compared with the control- (Fig. 7C). Furthermore, the endogenous BRs levels in the silenced lines were 8.7%–9.8% higher than in the TRV2-Mock (Fig. 7D). These findings suggest that inhibition of *BrWRKY8* expression in pakchoi leads to decreased transcription of *BrSGR2* and *BrCHI2*, along with increased BRs content, thereby delaying postharvest leaf senescence.

## Discussion

BRs are essential plant hormones that play a pivotal role in promoting plant growth and enhancing stress resistance [27]. EBR, a synthetic form of BRs, not only exhibits various physiological effects similar to BRs but also demonstrates higher activity. Even at low concentrations, EBR effectively regulates plant physiology and is nontoxic to humans [28]. Numerous studies have demonstrated that EBR can delay senescence and ripening in various vegetables and fruits [11–13, 29]. However, the mechanism underlying EBR’s delay of pakchoi leaf senescence remains unclear. To address this issue, we conducted transcriptome analysis of pakchoi treated with EBR and identified a key TF, *BrWRKY8*, which is both induced by senescence and regulated by EBR (Fig. 3A). This finding aligns with the known role of WRKY members in hormone-mediated leaf senescence [30].

During plant senescence, the expression of numerous TFs is upregulated, suggesting their involvement in the intricate regulatory network [17]. Notably, WRKY TFs play a crucial role in the plant senescence response [15]. However, while WRKY TFs have been extensively characterized in model plants, functional studies in nonmodel plants, particularly leafy vegetables, remain limited. In this study, we identified *BrWRKY8* through transcriptome analysis, which exhibited strong expression during late leaf senescence in pakchoi and was suppressed by EBR treatment. This expression pattern resembles that of *BrWRKY6* in Chinese flowering cabbage, which is regulated by GA [25]. These findings suggest that *BrWRKY8* may function as a regulatory element in the hormone-mediated network during leaf senescence in pakchoi. Multiple amino acid sequence alignments confirmed that *BrWRKY8* contains a conserved WRKY domain (Supplementary Fig. S5B) and exhibits typical characteristics of a TF. Subcellular localization and transcriptional activation analyses further corroborated that *BrWRKY8* localizes to the nucleus and possesses transcriptional activation activity (Fig. 3C-E). In conclusion, *BrWRKY8* is identified as a senescence-associated TF that is repressed by EBR in pakchoi.

Transcriptional regulatory network analysis has elucidated the significant role of WRKY TFs in regulating senescence in *Arabidopsis* [31]. Several *Arabidopsis* WRKY genes, including *AtWRKY6*, *AtWRKY42*, *AtWRKY45*, *AtWRKY53*, *AtWRKY55*, *AtWRKY71*, and *AtWRKY75*, have been identified as positive regulators of leaf senescence [15, 18, 19, 21, 31, 32]. Conversely, *AtWRKY70*, *AtWRKY57*, and *AtWRKY54* function as negative regulators of leaf senescence [20, 33]. In our study, we observed elevated expression of *BrWRKY8* transcripts in senescing leaves compared to other tissues (Supplementary Fig. S5E and F). This expression pattern resembles that of the established leaf senescence regulator *BrNAC55* in Chinese flowering cabbage [24]. To further elucidate the role of *BrWRKY8* in leaf senescence, we expressed *BrWRKY8* in *Arabidopsis* due to the limitations of the immature genetic transformation system in pakchoi. Overexpression of *BrWRKY8* in *Arabidopsis* induced premature leaf yellowing, accompanied with decreased Chl content and increased cell death in the leaves (Fig. 5A-D). To validate the function of *BrWRKY8* in pakchoi, we employed VIGS technology to silence the *BrWRKY8* gene. Notably, silencing *BrWRKY8* significantly delayed the leaf senescence process in pakchoi during storage, as evidenced by extended storage time, slower yellowing, and higher Chl content compared to controls (Fig. 7A and B). These findings strongly indicate that *BrWRKY8* positively regulates the senescence process in pakchoi.

Leaf senescence is a highly regulated process characterized by visible changes such as color alterations, wilting, and shedding [2]. It is influenced by various factors, including plant hormones that play a crucial role in its regulation [34]. Plant hormones often exert their effects by modulating the expression of TFs [21]. For example, the homologous gene of *BrWRKY8*, known as *AtWRKY8*, mediates both ethylene and ABA signaling in *Arabidopsis*, contributing to its defense response against the tobacco mosaic virus [35]. Additionally, *Arabidopsis* WRKY TFs such as *WRKY70*, *WRKY54*, and *WRKY46* have been implicated in the drought response and growth regulation controlled by BRs [36]. However, the regulatory mechanism of *BrWRKY8* in pakchoi senescence and its involvement in the EBR-mediated senescence regulatory network remain poorly understood. Our findings demonstrate that the expression level of *BrWRKY8* is significantly upregulated in senescent leaves and is suppressed by EBR treatment (Fig. 3B). Previous research by Zheng *et al.* revealed that overexpression of *MdWRKY9* in apple reduced BRs content, resulting in stunted seedling growth [37]. Correspondingly, our results indicate that overexpression of *BrWRKY8* in *Arabidopsis* results in lower BRs levels compared to wild-type plants, whereas *BrWRKY8*-silenced plants exhibit higher BRs levels (Figs 5E and 7D). Furthermore, we observed that EBR application inhibited the leaf yellowing caused by *BrWRKY8* overexpression (Fig. 6). Collectively, these results suggest that *BrWRKY8* may play a critical role in EBR-mediated senescence regulation in pakchoi.

WRKY TFs regulate plant senescence by modulating the expression of various downstream target genes. In *Arabidopsis*, *AtWRKY57* inhibits leaf senescence by suppressing *AtSEN4* and *AtSAG12* expression through the JA signaling pathway [20]. Conversely, *AtWRKY75* promotes leaf senescence by binding to *AtSID2* and *AtCAT2* promoters [19]. *AtWRKY45* directly regulates senescence-related genes, including *AtSAG12*, *AtSAG13*, *AtSAG113*, and *AtSEN4*, thus modulating leaf senescence [21]. Additionally, *AtWRKY71* promotes ethylene-regulated senescence by activating *AtEIN2*, *AtORE1*, and *AtACS2* expression [18]. In Chinese flowering cabbage, *BrWRKY65* promotes leaf senescence by regulating *BrNYC1*, *BrSGR1*, and *BrDIN* expression [25]. *BrWRKY6* regulates leaf senescence by binding to *BrSAG12*, *BrKAO2*, *BrNYC1*, *BrGA20ox2*, and *BrNYC1* promoters [24]. However, our understanding of WRKY TFs downstream target genes in pakchoi remains incomplete, with most WRKY family members’ target genes unknown. In this study, we demonstrated that *BrWRKY8* directly binds to *BrSGR2* and *BrCHI2* promoters through the ‘W-box’ element, activating their expression, using Y1H, ChIP-qPCR, and DLR experiments (Fig. 4A-D). In apple, *MdWRKY9* reduces BR production by inhibiting *MdDWF4* transcription, a BR-restricting enzyme [37]. Contrastingly, *BrWRKY8* may promote BRs degradation by enhancing *BrCHI2* expression. Notably, *BrWRKY8* overexpression in transgenic plants increased *AtCHI2* expression, a key gene in BRs degradation in *Arabidopsis*, while *BrWRKY8* silencing in TRV2-*BrWRKY8* plants suppressed *BrCHI2* expression in leaves (Figs 5F and 7C). Collectively, *BrWRKY8* may play a crucial role in EBR-mediated postharvest pakchoi leaf senescence by regulating *BrSGR2* and *BrCHI2* expression.

## Conclusions

**Figure 8:**
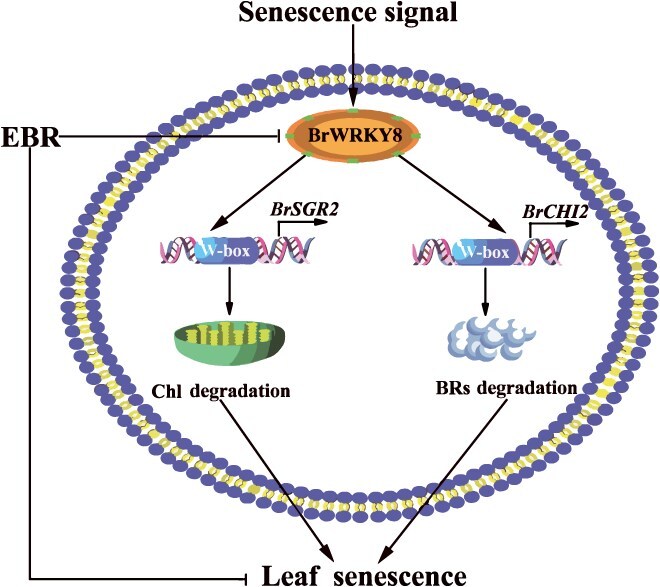
A proposed mechanism for *BrWRKY8* involvement in EBR-mediated delay of leaf senescence in postharvest pakchoi during storage

In conclusion, the EBR treatment effectively maintained BRs levels and delayed leaf senescence in pakchoi during storage. This study identified *BrWRKY8* as a crucial TF in the regulatory network responsible for EBR-induced delay of leaf senescence in pakchoi. These findings propose a working model for the function of *BrWRKY8* in the EBR-induced delay of leaf senescence in pakchoi (Fig. 8). The results of this research have significant implications for maintaining quality and improving storage resistance in leafy vegetables. Moreover, they provide valuable genetic resources for the molecular breeding of antisenescence vegetables.

## Materials and methods

### Plant materials and treatments

The pakchoi seeds utilized in this study were procured from the Vegetable Research Institute of the Jiangsu Academy of Agricultural Sciences and cultivated in the institute’s experimental field. The seedlings were harvested at approximately 40 days of age and promptly transported to the laboratory after precooling to eliminate field heat. Only pakchoi plants exhibiting uniform size and absence of mechanical damage or yellow leaves were selected for the experiment. The chosen pakchoi plants were treated with a 1.5 μM EBR (Sigma) solution, while the control group received distilled water. Each treatment group comprised three biological replicates, with each replicate containing 60 pakchoi plants. Following treatment, the pakchoi plants were placed in a culture box maintained at a temperature of approximately 20°C and a relative humidity of 80% in dark conditions.

*Arabidopsis* cultivation methods were implemented as detailed by Gao *et al.* [38].

### Chl analyses and surface color

To analyze Chl content, leaves from both *Arabidopsis* and pakchoi plants, collected from identical positions, were placed in 2-ml centrifugation tubes. Three biological replicates were utilized for each group. For Chl extraction, 1.5 mL of 80% pre-cooled acetone was added to each tube, ensuring complete leaf immersion. The sealed tubes were then incubated at 25°C for 20 h in darkness. Following the extraction period, light absorption values of the resulting solution were measured at wavelengths of 645 and 663 nm. Three technical replicates were conducted for each set of measurements.

Leaf surface color identification was performed according to the methodology outlined by An *et al.* [5].

### Endogenous BRs concentration assay

The crude enzyme extraction method employed in this study adhered to the protocol described by An *et al.* [5]. The procedure involved weighing 0.5 g of the sample, rapidly freezing it in liquid nitrogen, adding 5 mL of phosphate buffer, and thoroughly homogenizing the specimen manually or using a homogenizer. The specimen was then centrifuged at 4°C for approximately 14 min at 6000 *g*. Subsequently, the supernatant was collected. The BRs content was quantified using the BRs ELISA kit from Abmart (product code AB-4706B). After zeroing with a blank, the optical density (OD value) was measured at 450 nm using a plate reader. A standard curve was constructed by plotting the concentration of the standard on the horizontal axis and the OD value on the vertical axis. The concentration of plant BRs in the samples was then calculated using this standard curve.

### Plant total RNA extraction and qRT-PCR

The extraction of total RNA was conducted using the Vazyme (RC411–01) RNA extraction kit, while reverse transcription was performed using the TaKaRa one-step PCR amplification kit (AMV). Primers for qRT-PCR were designed utilizing the national center of biotechnology information (NCBI) online platform and underwent specific alignment (Supplementary Table S1). The qRT-PCR methodology followed the protocol described by Zhou *et al.* [39].

### RNA-sequencing

On days 0 and 4, total RNA was extracted from both the control and EBR treatment groups, with three biological replicates per group. The RNA samples were subsequently sent to Beijing Clouds Biotechnology company for the construction of nine transcriptome libraries and sequencing. The bioinformatics analysis was conducted following the methodologies described in previous studies from our laboratory [40].

### Subcellular localization of *BrWRKY8*

The subcellular localization method follows the protocol described by Liu *et al.* [41]. *Agrobacterium* harboring the *BrWRKY8*-GFP vector was cultured overnight to an optical density of 0.8. The bacteria were harvested by centrifugation at 5000 *g* for 10 min, discarding the supernatant. The bacterial pellet was resuspended in an equal volume of infiltration buffer (5 mM MgCl_2_, 8 mM MES, 90 μM Acetosyringone) and incubated for 3 h at room temperature in darkness. The bacterial solution was then mixed in equal proportions with the nuclear locus marker solution and injected into the abaxial surface of tobacco leaves using a needleless syringe. The inoculated tobacco was incubated at 25°C for 24 h, followed by 48 h in a light incubator at 25°C. Fluorescence signals were detected using a confocal laser microscope at 488 nm.

### Transcriptional activation assay in yeast

Yeast transformation methods are described with reference to Zheng *et al.* [37] The BD-*BrWRKY8* recombinant plasmid, BD, and BD-P53 plasmid were transformed into the Y2H Gold yeast strain. In this process, BD served as the negative control, while BD-P53 acted as the positive control. The transformation process adhered to the Y2H Gold yeast sensory instructions (Clontech). Subsequently, positive strains were identified, and cultured on selective medium (SD/-Trp-His-Ade). The growth of yeast strains was then observed and recorded.

### DLR assay

The promoter fragments of *BrSGR2* and *BrCHI2* genes were inserted into pGreen-0800-LUC vector to generate recombinant vectors. Subsequently, 35S::*BrWRKY8*, *BrSGR2*-pGreen-0800-LUC, and *BrCHI2*-pGreen-0800-LUC were transfected into *Agrobacterium tumefaciens* GV3101 and cultured in liquid medium containing kanamycin and rifampicin overnight until the OD600 value reached 0.8. The *BrSGR2*-pGreen-0800-LUC and *BrCHI2*-pGreen-0800-LUC bacterial suspensions were mixed with 35S::*BrWRKY8* bacterial suspension at a ratio of 1:9 (V/V), respectively. The mixed bacterial solution was then injected into tobacco leaves using a needleless syringe. The injected area (water-soaked area) was marked with a marker pen and incubated in a light chamber for 3 days. The enzymatic activities of firefly LUC and renilla REN luciferases were determined according to the kit instructions.

### *BrWRKY8* transformation into *Arabidopsis*

The *Arabidopsis* transgene methodology was based on the protocol described by Liu *et al.* [41]. Ten *Arabidopsis* wild-type plants, approximately 30 days old and well established, were prepared for transformation. To enhance transformation efficiency, flowers and fruit pods were removed two days prior to infestation, leaving only unflowering pods. *A. tumefaciens* containing the 35S::*BrWRKY8* construct was cultured overnight until reaching an OD600 of 1.5–2.0. The bacteria were harvested and resuspended in infiltration buffer (8% (W/V) sucrose, 0.01% (V/V) Silwet-77) to an OD600 of 0.8. The entire *Arabidopsis* inflorescences were submerged in the bacterial suspension for 1 min to ensure thorough transformation. Following infiltration, the *Arabidopsis* plants were dried, enclosed in a black plastic bag, and incubated in darkness for 24 h. Subsequently, the plants were uncovered and transferred to a light incubator for seed development. Transgenic seeds were harvested, and positive transformants were selected using hygromycin. Homozygous lines were obtained through continuous self-crossing for further analysis.

### Evans blue staining

Adapting the methodology of Haq *et al.* [42], *Arabidopsis* leaves at 35 days of age were completely submerged in a 0.35% (w/v) Evans blue solution for 24 h. Subsequently, the leaves were extracted and thoroughly rinsed with distilled water 3–4 times. After eliminating excess water, the leaves were heated in anhydrous ethanol:glycerin (9:1) for 30 min to extract Chl until the leaves became white. The leaf surface, now exhibiting visible blue spots due to Chl removal, was then flattened and photographed for analysis.

### Y1H assay

Initially, synthetic promoters of *BrCHI2* and *BrSGR2*, containing ‘W-box’ sequences, were inserted into the pAbAi vector as bait plasmids. The minimum concentration of AbA required to inhibit the growth of the integrated bait yeast was determined. Subsequently, the AD plasmid integrating the bait plasmid and the capture plasmid were transformed into a yeast strain. The transformed yeast strains were then plated on SD/-Leu/AbA plates and incubated in an inverted incubator at 30°C for 3 days. The AD+pAbAi-*BrCHI2* and AD+pAbAi-*BrSGR2* strains served as negative controls.

### ChIP assay

The ChIP methodology follows the protocol outlined by Liu *et al.* [43]. Briefly, pakchoi leaves were transfected with *A. tumefaciens* containing 35S::GFP and 35S::*BrWRKY8*-GFP plasmids, after which the leaves were harvested. Cross-linking was performed using 1.0% (v/v) formaldehyde at 25°C for 15 min, with the reaction subsequently quenched by adding glycine to a final concentration of 0.1 M. The chromatin was then sonicated and immunoprecipitated using an anti-GFP antibody. Sonication sheared the isolated chromatin to fragment sizes between 200 and 400 bp. DNA was extracted for use as a template in qPCR analysis. The PCR data were converted to CT values, calculated using input as a control (DNA purified by cross-linking directly after fragmentation, serving as the background of the material itself) to normalize the IP product, and input to normalize the product against the negative antibody-enriched product, immunoglobulin G (IgG). The difference in amplification cycles between IP and IgG after normalization was then calculated, and the fold enrichment was determined using the 2^-ΔΔCT^ algorithm.

### VIGS assay

The VIGS assay was conducted following the methodology described by Yue *et al.* [44]. A 300-bp specific sequence from the *BrWRKY8* coding region was inserted into pTRV2 to create the pTRV2-*BrWRKY8* fusion vector, which was then transferred into *Agrobacterium*. When wild-type pakchoi reached the three-true-leaf stage, 30 plants were inoculated with either pTRV1:pTRV2 (negative control) or pTRV1:pTRV2-*BrWRKY8* in a 1:1 (V:V) mixture. The mixture was injected into the abaxial surface of an appropriately sized true leaf using a needle-free 1-ml syringe. The roots were then cut and infused with the remaining fungal solution. Inoculated pakchoi plants were incubated in dark conditions at 28°C for 48 h. Subsequently, all plants were transferred to suitable growth conditions to monitor their growth status and morphological changes. After 2–3 weeks postinoculation, the replication and spread of pTRV2-*BrWRKY8* viruses in pakchoi leaves were assessed by semiquantitative PCR, while silencing efficiency was determined by qRT-PCR. Throughout this period, growth conditions and morphological changes were continuously monitored and recorded.

### Statistical analysis

The statistical analysis was conducted using Excel 2023 software, and the Statistical Package for Social Science 22.0 software was employed to ascertain significant differences (*P* < 0.05 indicates a significant difference).

## Supplementary Material

Web_Material_uhaf004

## Data Availability

The authors confirm that the data supporting the findings of this study are available within the article [and/or its supplementary materials].
