# Supplementary material for: BrWRKY8: a key regulatory factor involved in delaying postharvest leaf senescence of Pakchoi (Brassica rapa subsp. chinensis) by 2,4-epibrassinolide
Source: Hortic Res. 2025 Jan 6;12(4):uhaf004. doi: 10.1093/hr/uhaf004 (PMC11896971; doi:10.1093/hr/uhaf004)
Supplement: Web_Material_uhaf004 [file web_material_uhaf004.zip › Supplementary Figure.pdf]

Supplementary Information

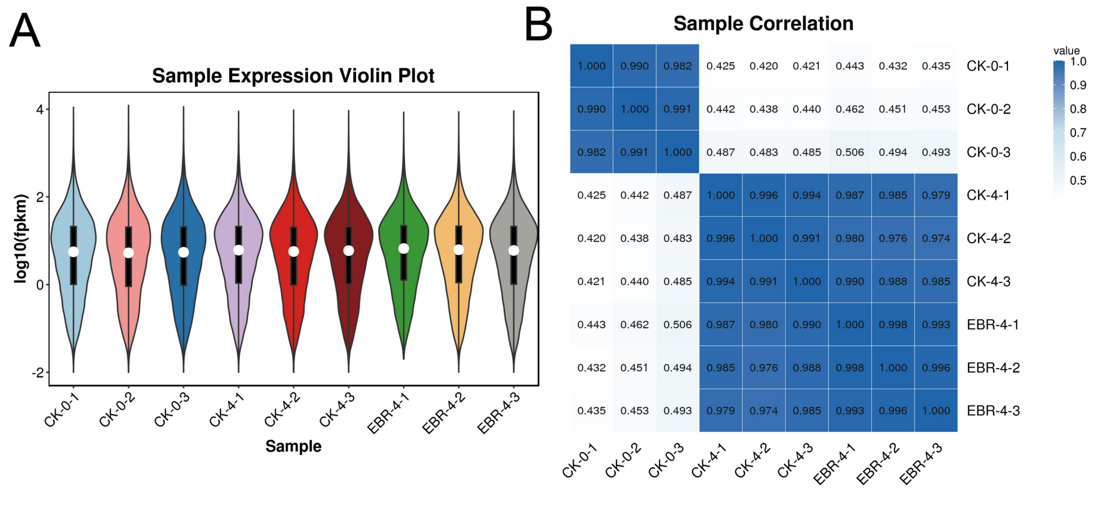

Figure S1. FPKM violin plot and heatmap showing correlation coefficient of the samples. (**A**) The FPKM distribution violin plot for each sample. The abscissa represents different samples; the ordinate indicates the logarithm of FPKM. (**B**) The correlation results for the samples.

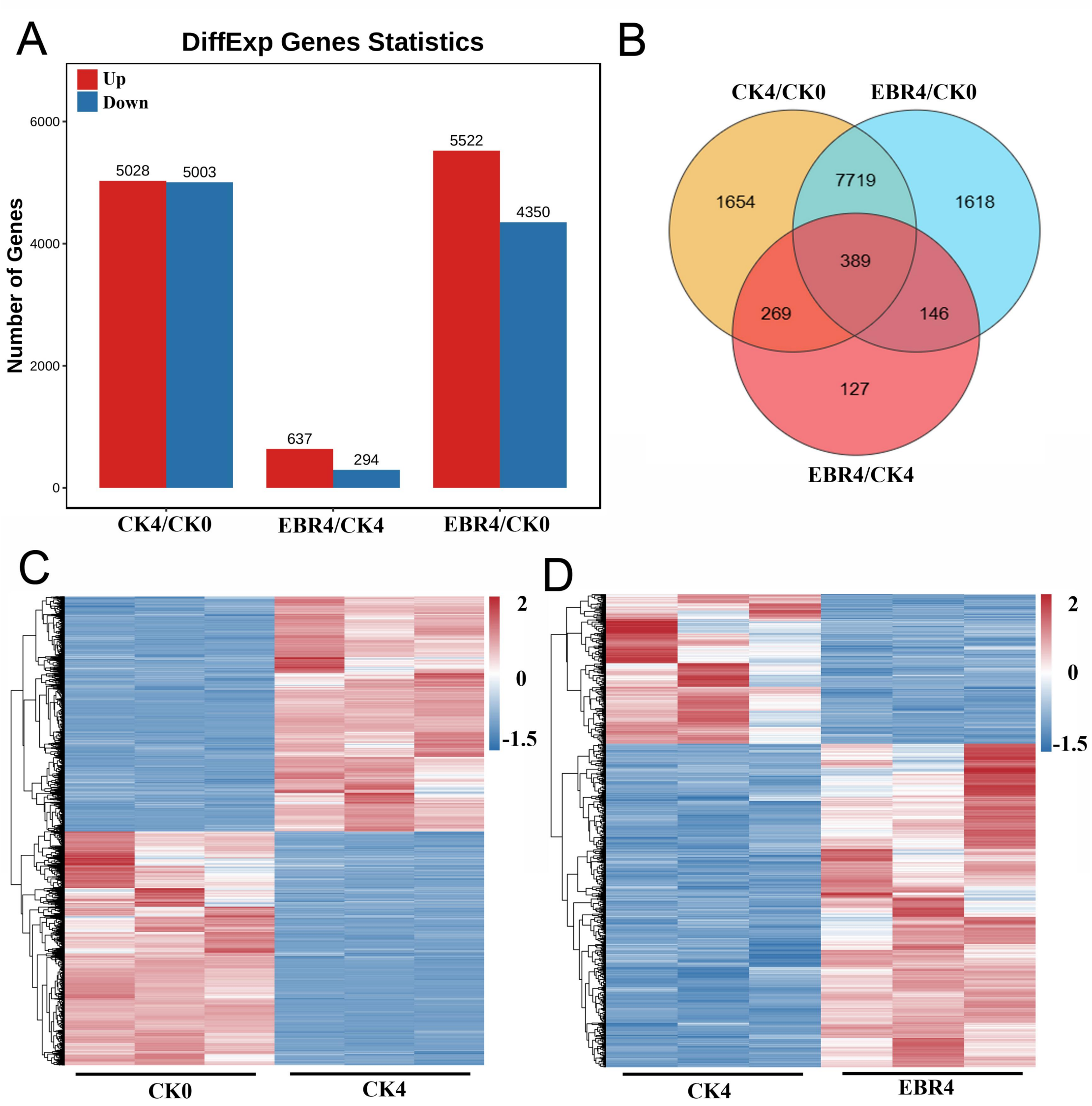

Figure S2. (**A**) Number of DEGs, (**B**) Venn diagram of DEGs (CK4/CK0, EBR4/CK0, and EBR4/CK4), (**C-D**) Heat map of DEGs (CK4/CK0 and EBR4/CK4).

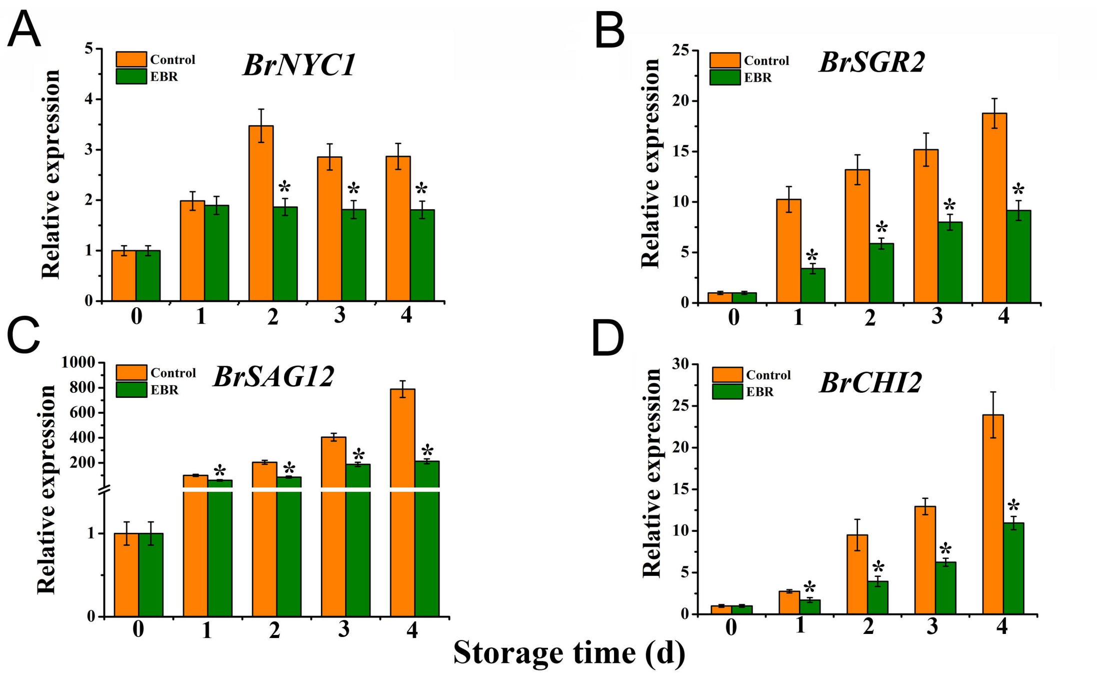

Figure S3. (**A-D**) Relative expression level of *BrNYC1*, *BrSGR2*, *BrSAG12*, and *BrCHI2* with and without EBR treatment during storage by qRT-PCR. Data represent means ± standard deviation of three biological replicates. Vertical bars indicate standard deviation. Asterisks denote significant differences at $P < 0.05$.

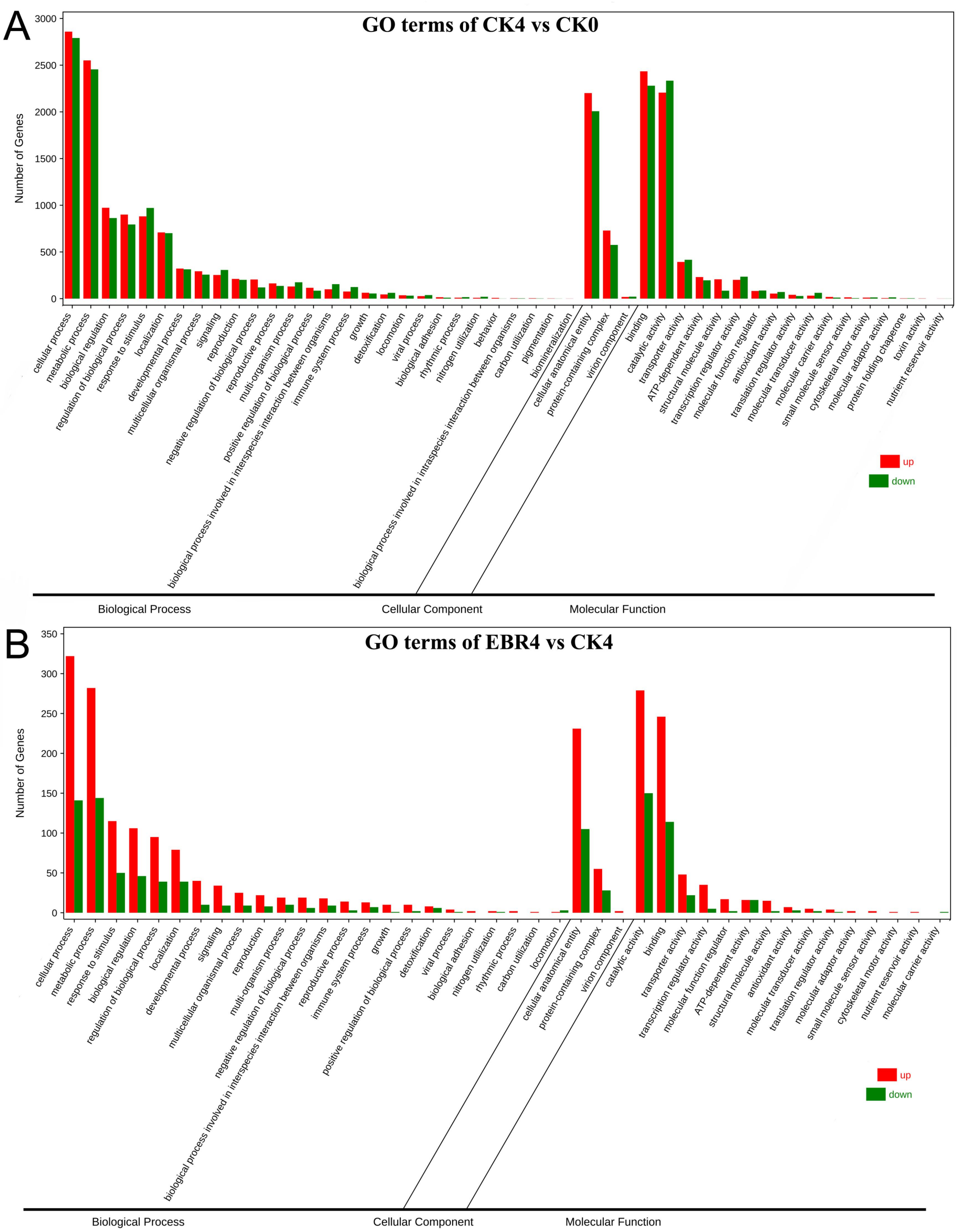

Figure S4. GO classification of DEGs in the CK4/CK4 (**A**) and EBR4/CK4 (**B**) group.

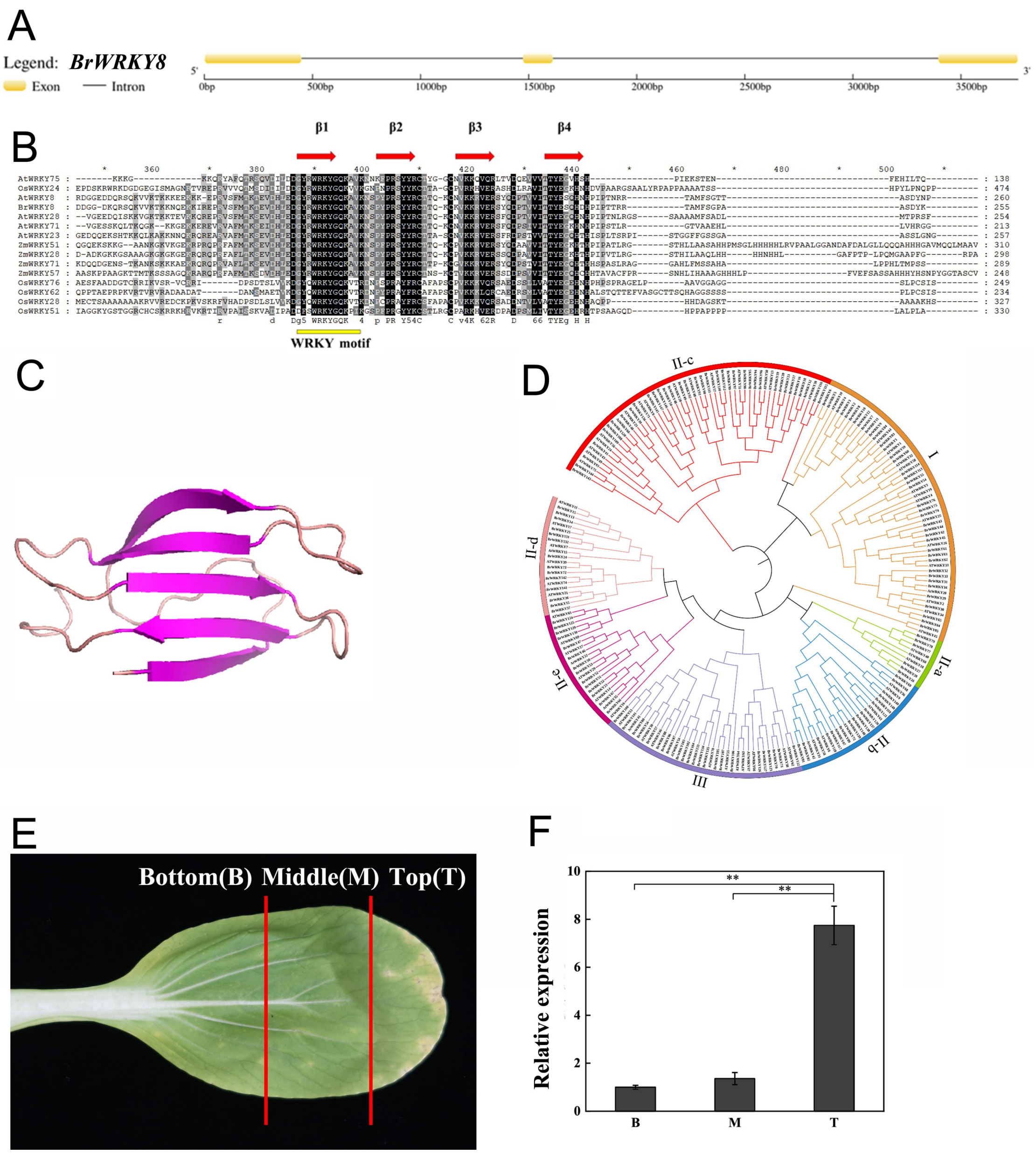

Figure S5. Basic information of *BrWRKY8*. (**A**) DNA structure of *BrWRKY8*. (**B**) Alignment of the amino acid sequences from *BrWRKY8* with that of its homologue WRKY from other species. (**C**) Predicted 3D protein model of *BrWRKY8*. (**D**) Phylogenetic analysis of pakchoi and *Arabidopsis* WRKYs genes. (**E**-**F**) Localized expression of *BrWRKY8* in a senescing leaf, Bottom (B) represents the non yellowing area, Middle (M) represents the early yellowing area, and Top (T) represents the yellowing area. Data represent means ± standard deviation of three biological replicates. Vertical bars indicate standard deviation. Asterisks denote significant differences at $P < 0.05$.

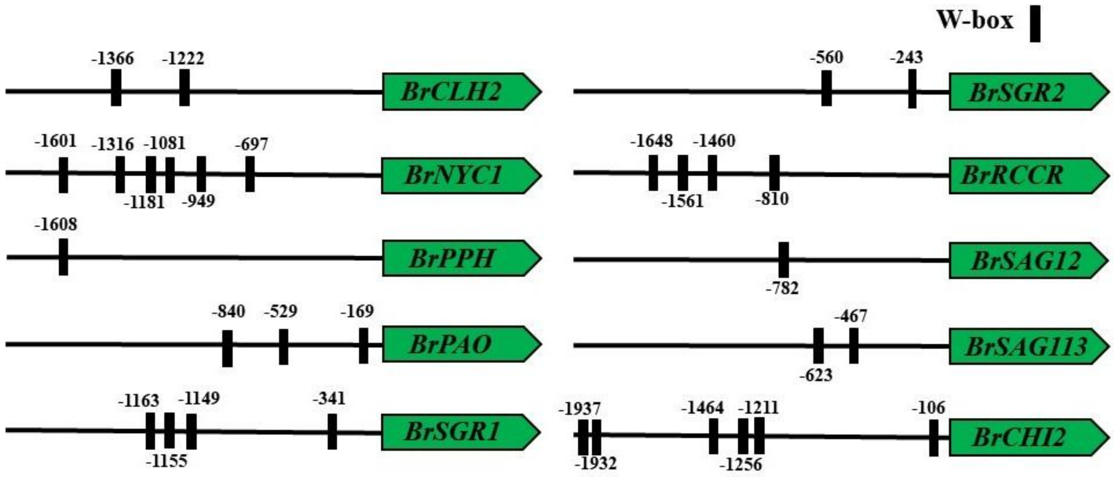

Figure S6. Schematic representation of *BrCLH2*, *BrNYC1*, *BrPPH*, *BrPAO*, *BrSGR1*, *BrSGR2*, *BrRCCR BrSAG12*, *BrSAG113* and *BrSGR2* promoter regions containing "W-box" clusters.

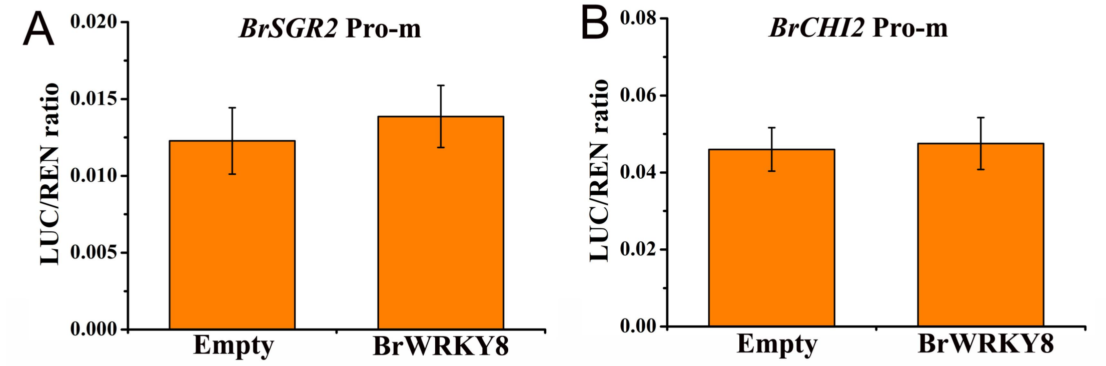

Figure S7. Interactions between *BrWRKY8* and promoters mutated (W-box core sequence ‘TGAC’ mutated to ‘AAAA’) at the binding site (*BrSGR2* pro-mutant / *BrCHI2* pro-mutant) were measured using DLR (A-B). Data represent means ± standard deviation of three biological replicates. Vertical bars indicate standard deviation.

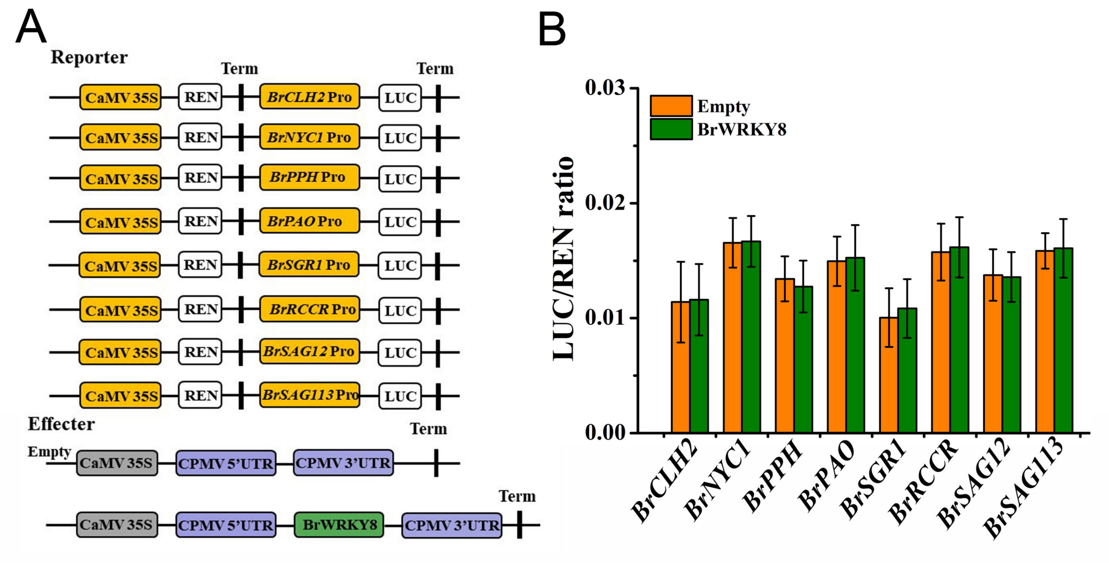

Figure S8. Interactions between *BrWRKY8* and promoters (*BrCLH2* pro, *BrNYC1* pro, *BrPPH* pro, *BrPAO* pro, *BrSGR1* pro, *BrRCCR* pro, *BrSAG12* pro, and *BrSAG113* pro) were determined using DLR, a schematic diagram of the vectors required for experimental analyses (A), and the LUC/REN ratio of *BrWRKY8* after co-expression of tobacco with downstream genes (B). Data represent means ± standard deviation of three biological replicates. Vertical bars indicate standard deviation.

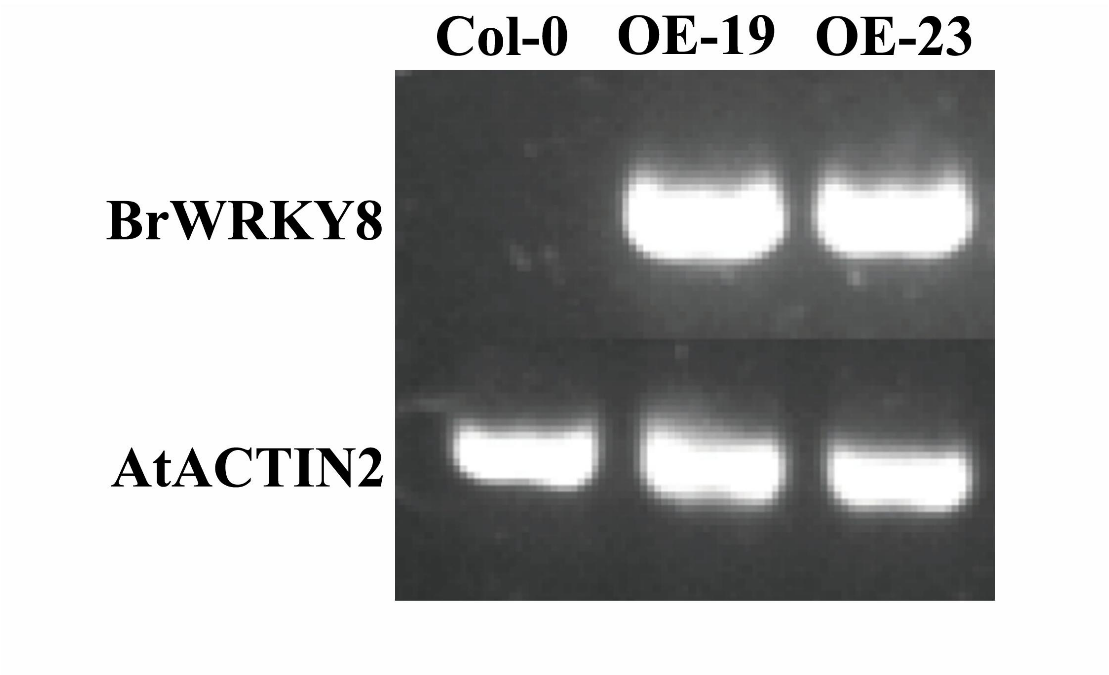

Figure S9. Semi-quantitative PCR analysis of *BrWRKY8* expression levels in Col-0, *BrWRKY8-OE19*, and *BrWRKY8-OE23*.

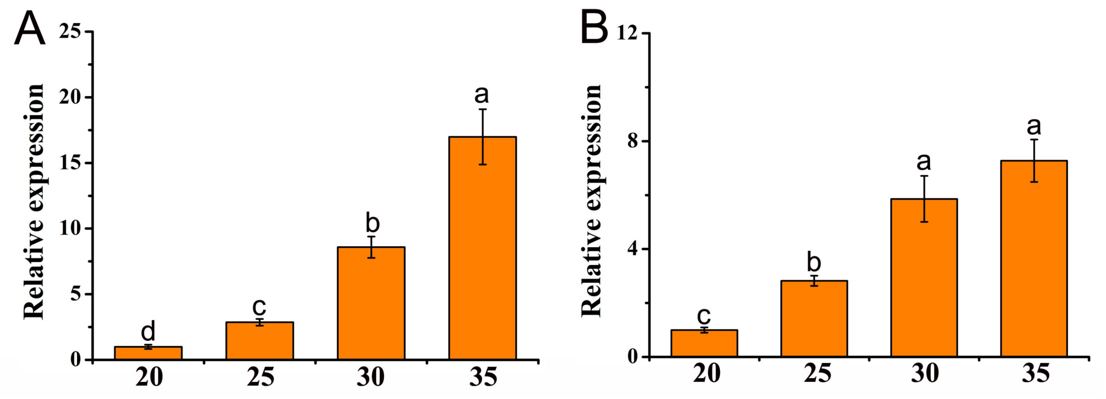

Figure S10. Determination of *AtSGR2* and *AtCHI2* expression in Col-0 at 25, 30, 35, and 40 days post-germination (A-B).Data represent means ± standard deviation of three biological replicates. Vertical bars indicate standard deviation. The distinct letters suggest significant differences at $P < 0.05$.

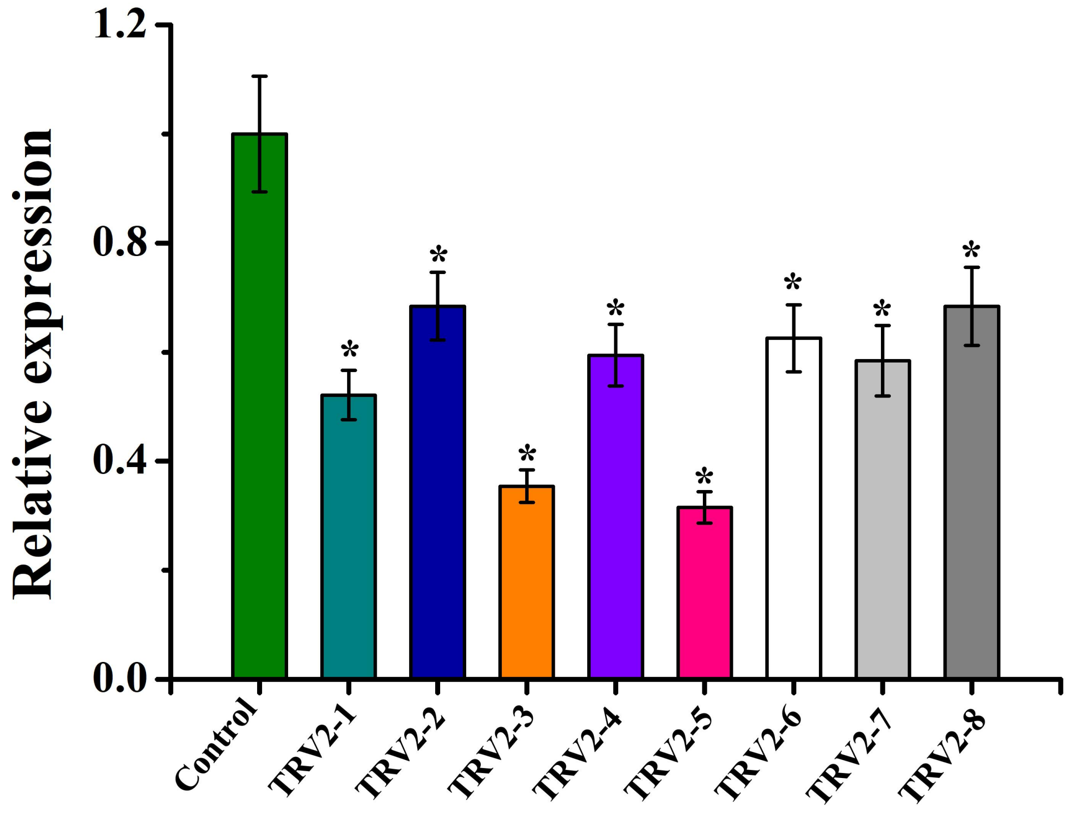

Figure S11. qRT-PCR analysis of transcriptional expression of *BrWRKY8* in VIGS lines. Data represent means ± standard deviation of three biological replicates. Vertical bars indicate standard deviation. Asterisks denote significant differences at $P < 0.05$.
